# The Therapeutic Management of Chemical and Herbal Medications on Uric Acid Levels and Gout: Modern and Traditional Wisdom

**DOI:** 10.3390/ph17111507

**Published:** 2024-11-09

**Authors:** Zhijian Lin, Jeetendra Kumar Gupta, Mohsin Maqbool, Krishan Kumar, Ayushi Sharma, Nitin Wahi

**Affiliations:** 1 Department of Clinical Chinese Pharmacy, School of Chinese Materia Medica, Beijing University of Chinese Medicine, Beijing 102488, China; linzhijian@bucm.edu.cn; 2 Department of Pharmacology, Institute of Pharmaceutical Research, GLA University Mathura, Chaumuhan 281406, India; 3 Department of Oncology, Dr. B. R. Ambedkar Institute Rotary Cancer Hospital, All India Institute of Medical Sciences, Ansari Nagar East, New Delhi 110029, India; 4 Department of Chemistry, Indian Institute of Technology Delhi, Hauz Khas, New Delhi 110016, India; 5 Institute of Molecular Biology, Academia Sinica, Taipei City 115, Taiwan; 6 Pathfinder Research and Training Foundation, Gr. Noida 201308, India; wahink@gmail.com

**Keywords:** gout management, hyperuricemia, phytochemicals, anti-inflammatory, herbal remedies

## Abstract

**Background:** Gout is a chronic inflammatory condition characterized by elevated uric acid levels in the blood, which can precipitate acute gout attacks in individuals with genetic susceptibility, existing medical conditions, and dietary influences. Genetic predispositions, comorbid medical conditions, nutritional choices, and environmental factors increasingly recognize the multifactorial etiology of the disease. **Methods:** Recent research has highlighted the potential of phytochemicals, particularly flavonoids, saponins, and alkaloids, to manage hyperuricemia (HUA) and its associated complications. **Results:** Plant’s natural compounds have garnered attention for their anti-inflammatory, antioxidant, and uric acid-lowering properties, suggesting their role in alternative and complementary medicine. Phytochemicals have demonstrated promise in mitigating gout symptoms and potentially modifying the disease course by addressing different aspects of hyperuricemia and inflammation. Herbal remedies, with their complex phytochemical profiles, offer a unique advantage by potentially complementing conventional pharmacological treatments. The integration of herbal therapies with standard medications could lead to enhanced therapeutic outcomes through synergistic effects, optimizing disease management, and improving patient quality of life. **Conclusions:** This review examines the current understanding of the multifaceted etiology of gout, explores the role of phytochemicals in managing hyperuricemia, and discusses the potential benefits of combining herbal remedies with conventional treatments to improve patient care and therapeutic efficacy.

## 1. Introduction

Gout constitutes one of the most prevalent types of inflammatory arthropathies. Research indicates a frequency of 1.7% in Australia and 2.7% in New Zealand, with elevated rates among Maori and islander communities [1]. The National Health and Nutrition Examination Survey (NHANES) in the United States and studies conducted in New Zealand, China, and the United Kingdom have shown a rising incidence of gout and hyperuricemia [2]. In 1965, research found no cases of gout among Aboriginal Australians; however, by 2002, the frequency had surged to 9.7% in males and 2.9% in females [3]. In the USA, the incidence of gout in 2007–08 was 6%, but hyperuricemia showed a frequency of 21% [4].

Hyperuricemia is characterized by serum uric acid levels above 0.36 mmol/L in females and 0.42 mmol/L in males [5]. Approximately 10% of individuals with hyperuricemia progress to gout, while 80–90% of gout patients exhibit hyperuricemia. The likelihood of getting gout rises with rising serum uric acid levels [6]. It is unclear why only a fraction of individuals with hyperuricemia develop clinical gouty arthritis [7]. Currently, there is inadequate evidence to endorse the treatment of asymptomatic hyperuricemia for the prevention of gouty arthritis, chronic renal disease, or cardiovascular events [8].

As discussed above, the historical data for gout showed a prevalence of hyperuricemia affecting more than one-fifth of the population in the USA [9]. It can be noted from the data presented that the Indigenous Australian population started at zero and reached an increase in prevalence to about 12.5% in 2002 [10]. Main factors such as genetics, diet, renal disease, and other comorbidities contribute to the variations in gout development among individuals with hyperuricemia [11]. Gout is ranked amongst chronic diseases that affect the quality of life, with acute flares resulting in pain and chronic disease resulting in inflammation, erosion of joints, and hence tophi [12]. Gout also leads to complex consequences, such as increased psychological tension, work-related injuries due to absenteeism, and increased healthcare expenses [13]. Despite the widespread application of efficient therapies, significant inconsistencies persist in diagnosing and managing gout due to variations in clinical recommendations, provider levels, and patient adherence to limited treatment [14]. The evidence does not support routine treatment of asymptomatic hyperuricemia to prevent gout or other complications [15]. However, the objective is to examine the therapeutic treatment of uric acid levels and gout from the viewpoints of modern medicine and traditional herbal knowledge. An examination of the effectiveness and safety profiles of various chemical and herbal therapies highlights the potential benefits of a comprehensive approach that combines scientific understanding with traditional methods. Comprehending the synergistic potential of various techniques may enhance the development of more tailored therapies for gout and elevated blood uric acid levels in clinical environments.

### Pathophysiological Mechanisms and Clinical Implications

Gout is a complex inflammatory arthropathy marked by the buildup of monosodium urate (MSU) crystals in joints and nearby tissues. This happens because of hyperuricemia, which means high uric acid levels in the blood [16]. The pathophysiological mechanisms of gout entail interconnected processes, wherein urate crystals precipitate in colder joint regions when serum uric acid levels surpass the solubility threshold, resulting in acute inflammatory responses [17]. The innate immune system plays a vital role in this process; the identification of MSU crystals by phagocytic cells, such as macrophages, activates the NLR inflammasome (NLRP1, NLRP3, NLRP6, NLRP7, NLRC4, NLRC5 and NAIP2/5/6), leading to the secretion of pro-inflammatory cytokines like interleukin-1β (IL-1β), which orchestrates the typical symptoms of gout, including severe joint pain, swelling, and erythema [18]. Epidemiological data reveal an increasing prevalence of gout, especially in developed nations, with notable associations between lifestyle factors—such as obesity, dietary patterns, and heightened intake of purine-rich foods and alcoholic beverages—and the occurrence of hyperuricemia, particularly among older males and those with comorbidities like hypertension and diabetes mellitus [19]. Considering the growing incidence of gout and its related comorbidities, it has become essential to address the clinical implications of these findings; the escalating occurrence and detrimental effects on patients’ quality of life demand a thorough comprehension of its pathophysiological foundations, as numerous existing treatment protocols are insufficient [20]. This study explores the relationship between lifestyle factors, serum uric acid levels, and gout incidence to improve clinical practice and develop targeted interventions. This will support public health initiatives aimed at reducing hyperuricemia and its complications, thereby addressing the urgent need for enhanced gout management strategies in clinical settings [21].

## 2. Etiology

The etiology of gout has become multifaceted, including genetic predisposition, medical comorbidities, and dietary influences [22]. Although most genetic anomalies cause only a small number of gout occurrences, most are characterized by increased blood uric acid concentrations, which can trigger clinical gout in predisposed individuals [23]. Heritability estimates for hyperuricemia and gout are around 73%, with 40–50% of patients reporting a familial history of the ailment [24].

**Figure 1:**
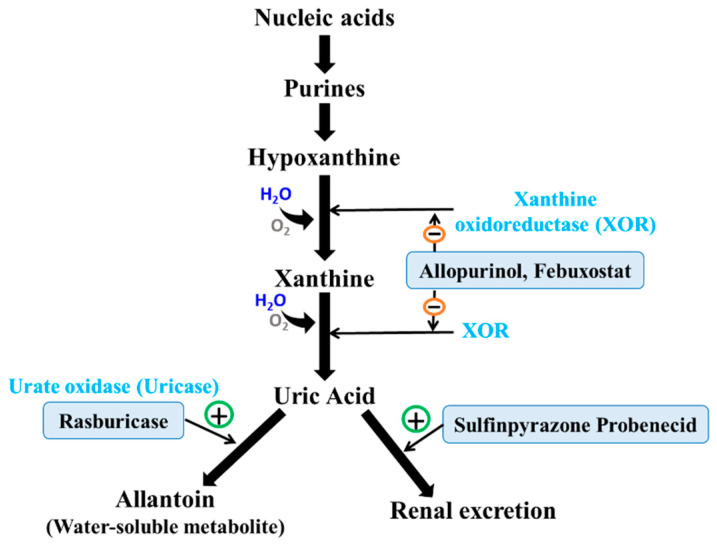
Uric acid production and metabolism. Main enzymatic targets and their drug inhibitors are shown to either inhibit (−) or enhance (+) uric acid synthesis and excretion.

**Figure 2:**
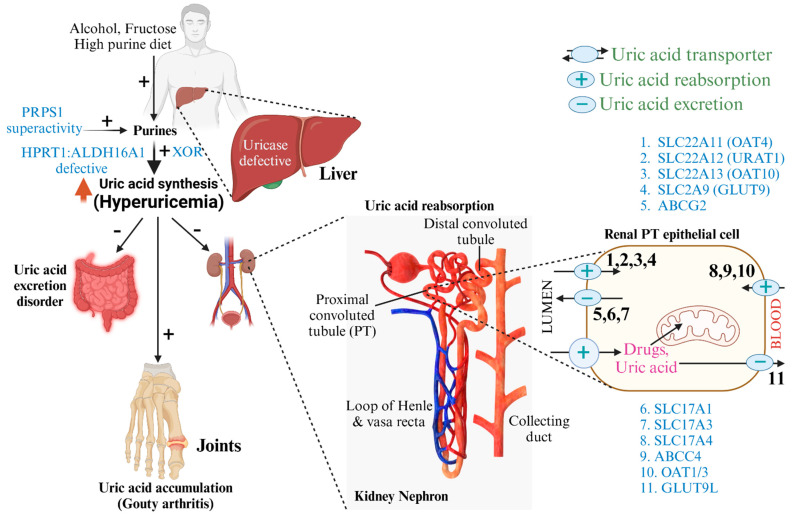
Mechanisms of uric acid production and excretion: Illustrates the biochemical pathways involved in uric acid metabolism, highlighting key enzymes and intermediates. Xanthine oxidase (XO) or XOR catalyzes the conversion of hypoxanthine to xanthine and, subsequently, to uric acid. Medications like allopurinol and febuxostat, XO inhibitors, reduce uric acid synthesis by inhibiting this enzyme, while rasburicase, a uricase analog, aids in uric acid breakdown. ALDH16A1 is a non-catalytic enzyme that causes gout via protein-protein interactions with HPRT1. OAT1/3 is situated on the basolateral membrane [29].

The familial clustering of gout suggests a genetic susceptibility linked to certain variations that might increase uric acid levels (Figure 1), resulting in repeated gout attacks [25]. Numerous essential genes in uric acid metabolism support this hereditary influence, offering prospective pathways for targeted therapeutic interventions [26]. The SLC22A11 (URAT1) and SLC2A9 (GLUT9) genes are crucial for the renal management of uric acid. The main job of URAT1 is to help uric acid be reabsorbed in the renal tubules. If this transporter is overactive, serum uric acid levels may rise, leading to hyperuricemia and gout [27]. Changes in the GLUT9 gene have been linked to altered uric acid transport processes, which show how complex genetic factors (Figure 2) are that affect the risk of getting gout [28].

Between URAT1 and GLUT9, the ABCG2 gene encodes a vital uric acid transporter that affects its excretion from the body [30]. Problems with ABCG2 or genetic variations could worsen hyperuricemia by making it harder for the body to get rid of uric acid, leading to gout [31]. The HPRT1 gene, essential for purine metabolism, influences uric acid levels via its involvement in the purine salvage route; disturbances in this process may result in elevated uric acid synthesis, exacerbating an individual’s risk for gout [32].

A highly significant element of gout pathophysiology pertains to the xanthine oxidoreductase (XOR) gene [33]. XOR is essential in purine catabolism, transforming hypoxanthine to xanthine and, thereafter, to uric acid. Elevated XOR activity can increase uric acid synthesis, contributing to hyperuricemia and markedly raising the risk of gout. This enzymatic pathway is essential since it establishes a direct connection between purine metabolism and uric acid buildup in the body [34]. Furthermore, the uricase gene, which encodes the enzyme that catalyzes the conversion of uric acid to allantoin, is also pertinent in gout. Humans have a nonfunctional version of the uricase gene due to evolutionary changes, leading to inefficient uric acid degradation [35]. This genetic modification results in a buildup of uric acid in the blood, predisposing them to gout attacks. Variants in XOR and uricase genes demonstrate the direct impact of genetic variables on an individual’s susceptibility to gout [36].

The complicated relationship between the genes URAT1, GLUT9, ABCG2, HPRT1, XOR, and uricase shows the complex genetic framework that controls the metabolism of uric acid and the risk of gout [37]. Comprehending these genetic effects improves our understanding of gout pathogenesis and reveals possible treatment targets for managing hyperuricemia and preventing gout flare-ups. This understanding may facilitate individualized therapy options that account for an individual’s distinct genetic composition, enhancing clinical results for individuals with gout [38].

Genes linked to gout are classified into four groups, as shown in Table 1. The concluding phase of purine metabolism is the conversion of hypoxanthine to xanthine and subsequently to uric acid by the action of xanthine oxidase, with allantoin produced from uric acid by uricase [39]. However, certain animals, such as humans, lack uricase owing to genetic changes around 25 million years ago. Simultaneously, increased URAT1 activity was developed to improve uric acid excretion. The cessation of vitamin C synthesis in humans and other primates some 20 million years ago has prompted the notion that uric acid originated as an alternative antioxidant [40]. Dietary habits substantially affect the likelihood of getting gout, a disorder marked by increased blood uric acid levels. Elevated urate concentrations significantly increase the risk of gout, with studies showing that patients with heightened levels encounter more frequent exacerbations [41]. A study of nearly 2000 older people revealed that individuals with blood urate levels over 9 mg/dL were three times more likely to suffer a gout flare within 12 months than those with levels below 6 mg/dL [42]. It is essential to recognize that not all patients with hyperuricemia will exhibit gout. The intake of high-purine meals, including shellfish and organ meats, in conjunction with liquids abundant in fructose or alcohol, leads to hyperuricemia and gout. Epidemiological studies indicate a growing prevalence of gout associated with modern lifestyle modifications, such as heightened protein consumption and reduced physical activity [43]. Contributing factors encompass old age, male gender, obesity, and the administration of drugs, including diuretics and low-dose aspirin [44]. Significant consumption of purine-laden foods, such as red meat and seafood, is associated with a 50% elevation in gout risk for people consuming more than 24 ounces weekly, in contrast to those ingesting fewer than 12 ounces [45]. Furthermore, the consumption of sugary drinks and excessive alcohol, particularly beer, raises blood uric acid levels, with each additional 12 ounces of beer consumed monthly linked to a 20% increase in gout risk [46]. Lifestyle factors, such as physical inactivity, significantly contribute to the risk of gout. A study demonstrates that individuals with low physical activity levels have a 30% higher probability of acquiring gout than their more active peers [47]. Obesity significantly heightens this risk, as studies demonstrate that every five-unit increase in body mass index (BMI) correlates with a 38% increase in the incidence of gout [48,49]. Moreover, specific drugs, particularly diuretics, may exacerbate gout, with thiazide diuretics associated with a 60% increased risk of getting the condition compared to non-users. Medications such as low-dose aspirin can additionally influence uric acid levels, thus compounding gout’s risk profile [50].

## 3. Pathophysiology

The liver primarily synthesizes uric acid, a key end product of purine catabolism, through the enzymatic breakdown of purines in various foods and nucleic acids [51]. The enzyme xanthine oxidase (XO) is involved in this process. It speeds up the changes from hypoxanthine to xanthine and uric acid. Uric acid serves an important role as an antioxidant, helping to neutralize free radicals and protect the body from oxidative stress. However, excessive uric acid levels in the bloodstream can lead to hyperuricemia, increasing the risk of gout—an inflammatory arthritis characterized by painful urate crystal deposits in the joints. Medication targets various points in this metabolic pathway. Allopurinol and febuxostat, for example, inhibit XO to reduce uric acid synthesis, while rasburicase, an uricase analog, facilitates the breakdown of uric acid into more soluble forms [49,50]. Drugs like sulfinpyrazone and probenecid also enhance the renal excretion of uric acid by inhibiting its reabsorption in the renal tubules, effectively lowering blood uric acid levels (Figure 1).

In therapeutic contexts, medications target various points in this metabolic pathway. In individuals, the lack of the enzyme uricase, present in most mammals and responsible for converting uric acid into the more soluble allantoin, leads to increased urate concentrations in the bloodstream (Figure 1). Renal excretion primarily excretes uric acid, accounting for around two-thirds of total excretion, with the gastrointestinal system excreting the remaining one-third. Under typical physiological settings, serum uric acid levels are sustained between 3.5 and 7.2 mg/dL; however, hyperuricemia occurs when uric acid production surpasses excretion. Excessive production associated with dietary influences, disorders like psoriasis, or compromised excretion due to renal illness or specific drugs can cause this syndrome [52,53,54,55,56,57].

Monosodium urate (MSU) crystals can form in the joints and other tissues when someone has chronic hyperuricemia. These crystals cause acute inflammation. The NLRP3 inflammasome, an essential element of the innate immune system, becomes active upon detecting MSU crystals by pattern recognition receptors on immune cells. This activation induces the formation of the NLRP3 inflammasome complex, consisting of NLRP3, ASC, and procaspase-1, which subsequently activates caspase-1. Active caspase-1 cleaves the pro-inflammatory cytokines pro IL-1β and pro IL-18 into their active forms, releasing and recruiting extra immune cells and enhancing the inflammatory response [58]. NLRP3 activation also causes pyroptosis, a programmed cell death (PCD, apoptosis) that worsens local inflammation and may keep the inflammatory cycle going, affecting the whole body [59].

Chronic hyperuricemia and the resultant deposition of monosodium urate crystals lead to the formation of tophi, pathological characteristics of advanced gout. Initially asymptomatic, these deposits can evolve into palpable, hard nodules that apply pressure on adjacent structures, leading to inflammation and discomfort [60]. The inflammatory milieu created by tophi exacerbates the deterioration of joint tissues, resulting in the obliteration of articular cartilage and subchondral bone. This approach ultimately leads to considerable joint degeneration and abnormalities. Tophi signifies a more advanced phase of gout, requiring intensive treatment approaches focused on reducing uric acid levels and controlling inflammation. Not paying enough attention to these things can cause long-lasting pain and problems with daily life. This shows how important it is to find and treat hyperuricemia as soon as possible to avoid permanent damage to joints and other issues that can come with it, like heart disease and kidney stones [29,61,62,63].

## 4. Modern Pharmacological Management of Gout

Gout is an instance of inflammatory arthritis resulting from increased uric acid levels in the bloodstream, a condition referred to as hyperuricemia [61]. Excessively elevated uric acid levels could result in the formation and deposition of monosodium urate crystals in joints, causing severe discomfort, swelling, and inflammation [62]. Often, these deposits appear in joints like the big toe, but they can also affect other areas like the knees, ankles, and wrists. The resulting ailment is referred to as gouty arthritis. The prognosis of gout centers has two main objectives: controlling acute inflammatory episodes and sustaining long-term regulation of uric acid levels to avert recurring attacks. Treatment techniques have progressed markedly in recent years [63]. During an acute gout episode, the primary objective is to alleviate inflammation, often accomplished with anti-inflammatory agents, such as nonsteroidal anti-inflammatory drugs (NSAIDs), corticosteroids, or colchicine. Long-term therapy aims to reduce uric acid levels to prevent the formation of new crystals and reduce the likelihood of recurring flare-ups. This is often achieved with urate-lowering medications such as allopurinol and febuxostat. Recent advancements in gout care include the emergence of novel biological medicines, for instance, when gout is unresponsive to standard therapies [64]. These biologics specifically target pathways associated with inflammation and immune response and provide supplementary therapy alternatives for patients with refractory gout, enhancing results in more severe instances [65].

### 4.1. Chemical Medications for Uric Acid Control

#### 4.1.1. Xanthine Oxidase Inhibitors

**Figure 3:**
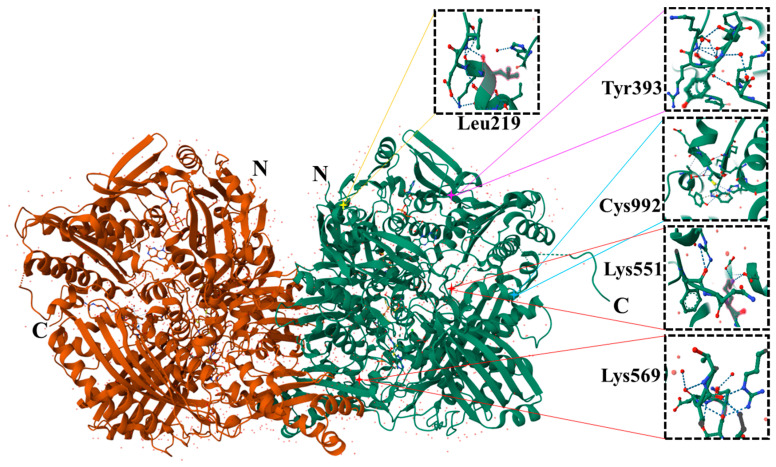
The crystal structure of bovine milk xanthine dehydrogenase (XDH dimer with three primary domains and two connecting loops) highlights bound NAD (color-coded for clarity, downloaded from PDB). The N to C terminus domains are Fe-S cluster, FAD, and molybdopterin (Mo-pt). Residues 192–225 link the iron-sulfur clusters to the FAD domain, whereas residues 537–589 connect the FAD domain to the Mo-pt domain. The N and C termini are indicated. This includes the FAD cofactor, two iron-sulfur, the molybdopterin cofactor, and salicylate. Leu 219 and Lys 569 are in the flexible connector segments of the FAD domain. Tryptic proteolysis of XDH after Lys 551 or pancreatin cleavage after Leu 219 and Lys 569 will result in irreversible transformation to XO. The right panel displays structural fragments that indicate bound inhibitors and specific amino acid residues essential for enzyme functioning, including the covalently bound oxypurinol inhibitor. The color-coded stars (yellow—leucine, magenta—tyrosine, cyan—cysteine, and red—lysine) indicate the well-defined positions of crucial amino acids in 3D folded proteins for stable interactions mentioned elsewhere by Enroth C et al. (PNAS. 2000;97(20):10723-8).

Xanthine oxidase (XO, EC 1.1.3.22) and xanthine dehydrogenase (XDH, EC 1.17.1.4) are alternative forms of xanthine oxidoreductase (XOR), a molybdopterin-containing flavoprotein composed of two identical 145 kDa subunits as shown in Figure 3 [66]. Mammals produce XOR in the dehydrogenase form, but sulfhydryl oxidation or proteolysis can transform it into the oxidase form. XO and XDH exhibit structural similarities, including congruent X-ray absorption spectra, and are classified within the molybdenum hydroxylase family [67]. Recent mechanistic insights, primarily obtained from crystallographic investigations of XOR and aldehyde oxidoreductase, have clarified the enzyme’s catalytic mechanism, whereby hydroxylation of xanthine transpires at the molybdopterin core, followed by electron transfer to several redox centers [68]. Each XOR subunit autonomously promotes catalysis, characterized by a structure including [Fe-S] clusters, a FAD domain, and a molybdopterin-binding domain [69]. These studies have enhanced the comprehension of the structure-function links of XOR.

➢ Allopurinol and Oxypurinol

Allopurinol and febuxostat are the primary xanthine oxidase inhibitors (XOIs) used to reduce uric acid production by targeting xanthine oxidase (Figure 3), the enzyme responsible for converting hypoxanthine to xanthine and subsequently to uric acid [70]. Allopurinol, a cornerstone in gout treatment since its FDA approval in 1966, works by inhibiting xanthine oxidase and reducing serum uric acid levels. However, it requires dose adjustments in patients with renal impairment and carries the risk of hypersensitivity reactions such as the rare allopurinol hypersensitivity syndrome (AHS) [71,72].

**Figure 4:**
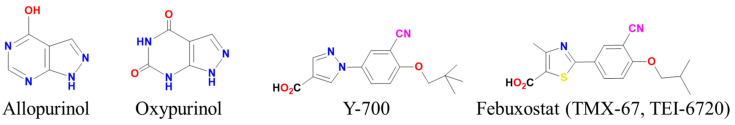
Chemical structures of selected xanthine oxidase inhibitors [74].

Febuxostat, a newer and more selective XOI, is particularly useful for patients intolerant to allopurinol but is associated with an increased risk of cardiovascular events, necessitating careful monitoring [73]. Both medications effectively lower serum urate levels but differ in side effect profiles, including gastrointestinal discomfort and rash with allopurinol, liver function abnormalities, and joint pain with febuxostat [72]. The active metabolite of allopurinol, oxypurinol (Figure 4, Table 2), has prolonged pharmacological effects due to its long persistence in tissues.

#### 4.1.2. Uricosuric Agents

Uricosuric agents are a class of medications aimed explicitly at treating hyperuricemia, a condition characterized by elevated levels of uric acid in the blood, which can lead to gout [70]. These agents promote uric acid excretion through the kidneys. Their primary action mode involves inhibiting uric acid reabsorption in the renal proximal tubules, where uric acid is typically filtered and reabsorbed into the bloodstream [77]. By targeting the URAT1 (urate transporter 1) protein, an essential transporter responsible for the reuptake of uric acid, uricosuric agents effectively block this process, allowing more uric acid to be excreted through urine [78]. This reduction in serum uric acid (SU) levels helps to prevent the crystallization of uric acid in joints and tissues, which is a major cause of the painful inflammation associated with gout [79]. By maintaining lower uric acid levels, these medications help manage both acute gout flares and chronic hyperuricemia, reducing the risk of long-term joint damage and other complications associated with the disease [56].

#### 4.1.3. Conventional Approaches to Gout Management

Gout is a type of arthritis that results from the deposition of monosodium urate in the joints, causing inflammation [76]. Lifecycle management of the disease primarily focuses on using the sun, which helps counter the inflammatory response sooner rather than later and eases pain [80]. Recommendations from the American College of Rheumatology (ACR), the European League Against Rheumatism (EULAR), the British Society of Rheumatology (BSR), and the American College of Physicians (ACP) Clinical Practice Guideline for the Management of Rheumatoid Arthritis suggest using non-steroidal anti-inflammatory agents (NSAIDs), colchicine, or corticosteroids as first-line therapies [81]. Understanding these sentiments influences treatment, such as timing, choosing the correct medications, and even addressing other illnesses [82].

➢ Non-steroidal anti-inflammatory drugs (NSAIDs)

Short-lived NSAIDs, such as indomethacin, naproxen, and ibuprofen, are commonly prescribed for the management of acute gout crises [83]. These nonsteroidal anti-inflammatory drugs (NSAIDs) function primarily by inhibiting cyclooxygenase (COX) enzymes, which are crucial for the biosynthesis of prostaglandins—**key** mediators in the inflammatory response [84]. By blocking COX enzymes, NSAIDs effectively reduce the pain and inflammation associated with acute gout attacks.

Despite their efficacy, prolonged use of NSAIDs is associated with several adverse effects [85]. These include gastrointestinal complications such as ulcers, bleeding, renal impairment, and an increased risk of cardiovascular events. The risk of these adverse effects is particularly pronounced in elderly patients and individuals with preexisting health conditions [86]. Thus, while NSAIDs are effective for short-term relief of gout symptoms, careful consideration of their potential risks is essential, especially in long-term management and in vulnerable populations [87].

➢ Colchicine

Colchicine has been used to treat acute gouty arthritis for many years. In this regard, it is considered adequate due to its inhibiting the polymerization of microtubules, preventing neutrophils from migrating to the site of inflammation, thereby reducing the presence of urate crystals. It has been explained that colchicine should be taken at the onset of an attack to yield great results. However, there is a risk of a narrow therapeutic margin and severe side effects, including gastrointestinal effects like nausea, vomiting, and diarrhea. Lower-dose regimens are now more practiced to lessen the toxicity of the drug. In addition to bone marrow suppression and myopathy, a pre-existing renal or hepatic disease can also trigger or exacerbate the toxicity of colchicine [88,89,90,91,92,93,94].

➢ Biologic materials

In patients with refractory gout who do not respond to treatment with conventional drugs, supportive options are needed, including the use of biologics that inhibit selected inflammatory mediators of inflammation [95]. In this setting, therapy with IL-1 inhibitors and uricase-based therapies reduced inflammation and high uric acid levels in patients with conventional therapy failure. Interleukin-1 (IL-1) is one of the most potent mediators of inflammation and drives painful attacks of gout as part of the immune reaction to masses of monosodium urate (MSU) crystals [96]. Anakinra IL-1 receptor antagonist has been shown, in patients with chronic gout whose diseases were unresponsive to standard treatment, to suppress pain and inflammation by inhibiting IL-1 action [97]. Most patients will tolerate anakinra, but it comes with a potential risk of infections at the injection site and limitations to its general use because of other side effects [98]. Canakinumab is another IL-1 inhibitor studied to decrease flare-ups, particularly in the severe forms of gout. However, the efficacy of the drug combined with its potential side effects leads to the use of this drug in patients with severe resistant gout [99].

### 4.2. Traditional Herbal Remedies for Gout Treatment

Gout, a form of metabolic arthritis that results from acute mono sodium urate (MSU) crystal deposition, has completely different treatment approaches from those used in Western medicine and those used in Ayurvedic and TCM practices [100]. They serve as historical evidence for the worth of using natural preparations for the treatment management of gout, especially if the standard therapies such as colchicine, corticosteroids, and non-steroidal anti-inflammatory drugs (NSAIDs) prove ineffective because of their side effects like dyspepsia, kidney disease, and failure to provide progression of disease management [101].

Gout in the Ayda system (in Ayurveda, referred to as Vatarakta) is addressed by changing one’s regular diet and administering herbal medicines to control the dose, as well as increasing the metabolism of the body and excretion of the wastes. Such herbs as Guduchi (*Tinospora cordifolia*), Guggulu (*Commiphora wightii*), and Shallaki (Boswellia serrata), which are all anti-inflammatory and uric acid-depleting agents, are commonly used [81].

#### 4.2.1. Traditional Chinese Herbal Medicine

Traditional Chinese medicines (TCMs), such as Jiawei Simiao powder (JWSMP), Jinhuang cream, and various decoctions like Tongfeng, Danxi Tongfeng, Wuwei Xiaodu, Zhuye Shigao, Qingre Chubi, Tongyang Mizhuo, Quzhuo Tongbi, and capsules like Tongfenshu, each employ distinct mechanisms to treat and alleviate gout symptoms [102]. For instance, Zhuye Shigao decoction (which includes *Folium Phyllostachydis Henonis*, *Gypsum Fibrosum*, *Rhizoma Pinelliae Praeparatum*, *Radix Ginseng*, *Radix Ophiopogonis*, *Oryza Sativa*, *Radix Ophiopogonis Japonicir*, and *Radix et Rhizoma Glycyrrhizae*) has been shown to reduce serum levels of IL-1β and caspase-1 and downregulate the expression of pro-IL-1 and pro-caspase-1. This suggests that Zhuye Shigao decoction mitigates inflammation caused by sodium urate crystals by interfering with the IL-1 signaling pathway [103]. Comparatively, Chinese herbal medicines have demonstrated superior anti-inflammatory effects relative to conventional therapies in 19 trials involving 17 different prescriptions and are generally safer. However, no significant difference in clinical efficacy was observed between herbal and Western medicines during acute gout attacks, based on parameters such as C-reactive protein, serum uric acid, erythrocyte sedimentation rate, and overall clinical response [104]. Notably, Chinese herbal medicines often showed comparable efficacy with fewer adverse drug reactions (RR: 0.06, 95% CI: 0.03 0.13).

#### 4.2.2. Malaysian Medicinal Plants

In a study investigating antigout potential through the inhibition of xanthine oxidase (XO), eighty-five plants were identified, with notable findings on their efficacy [105]. *Momordica charantia* demonstrated the highest XO inhibitory activity, achieving 96.5% inhibition at 100 μg/mL, making it the most potent among the Cucurbitaceae family. In the Zingiberaceae family, *Kaempferia galanga* exhibited the highest XO inhibitory activity, followed by Zingiber officinale (81.56%), *Alpinia galanga* (57.99%), and *Curcuma longa* (28.31%) [106]. Additionally, plants from the Asteraceae family, including *Artemisia vulgaris, Blumea balsamifera, Chrysanthemum indicum*, and *Chrysanthemum sinense*, were evaluated, and *Chrysanthemum indicum* showed the highest XO inhibitory activity at 95%. These variations in efficacy are attributed to the diverse nature of bioactive compounds such as flavonoids, phenolics, tannins, coumarins, luteolin, and apigenin present in these plants [107].

### 4.3. Potential Drug-Herb Interactions

The concurrent application of herbal treatments and conventional drugs necessitates careful examination of potential drug-herb interactions [108], as they can greatly affect patient safety and therapeutic efficacy, as illustrated in Table 3. Certain herbs may influence the pharmacokinetics of non-steroidal anti-inflammatory drugs (NSAIDs) or colchicine, potentially causing increased side effects or reduced therapeutic efficacy; for instance, herbs that inhibit cytochrome P450 enzymes could modify the metabolism of these drugs, leading to elevated plasma concentrations and an increased risk of toxicity [109]. Moreover, herbal medicines may have specific contraindications, especially in people with preexisting health disorders like liver or kidney illness, which might increase the risk of unpleasant responses [110]. This interaction requires thorough patient evaluations, encompassing an exhaustive medication history and an assessment of the patient’s health condition, to guarantee that the integrated therapy strategy is both safe and effective. Due to the increasing interest in complementary and alternative medicine, healthcare providers must stay alert to these interactions, promoting a collaborative strategy that integrates conventional and herbal therapies while prioritizing patient safety and enhancing therapeutic results [111].

### 4.4. Phytochemicals and Their Potential in Uric Acid Reduction

Phytochemicals, particularly flavonoids, saponins, and alkaloids, have generated significant attention in natural therapy for their potential to address hyperuricemia (HUA) and associated health issues, as shown in Table 4 [123]. Hyperuricemia, defined by increased serum uric acid concentrations, may result in gout and many metabolic problems [124]. This section analyzes the function of these phytochemicals in lowering uric acid levels, emphasizing their processes and therapeutic uses for treating hyperuricemia [125].

## 5. Synergistic Effects of Combining Herbal and Chemical Treatments

A prevailing trend in contemporary healthcare is the integration of modern and traditional therapeutic approaches, recognizing the added value derived from combining herbal remedies with conventional medications [127]. This integrative strategy enhances patient care by improving therapeutic efficacy and mitigating the toxicity often associated with standard treatments [128]. Herbal medicine, extensively utilized in Ayurvedic and traditional Chinese medicine (TCM) systems, offers a range of pharmacological effects, including anti-inflammatory, antioxidant, and immunomodulatory activities [129]. These remedies can augment the effects of conventional drugs or serve as alternatives. For instance, traditional management of gout—a condition characterized by the deposition of uric acid crystals in the joints—typically involves nonsteroidal anti-inflammatory drugs (NSAIDs) and urate-lowering agents such as allopurinol [130]. However, these medications are frequently associated with adverse effects, including gastrointestinal disturbances and increased risk of organ toxicity, particularly with prolonged use. The addition of herbal remedies such as *Terminalia bellerica*, which reduces uric acid and inflammation; *Zingiber officinale* (ginger), which alleviates pain and swelling; and *Allium sativum* (garlic), which also decreases swelling and uric acid levels, can provide supplementary therapeutic benefits [131].

These herbs could enhance the efficacy of conventional treatments by further lowering uric acid levels and reducing inflammatory responses, thereby improving patient outcomes and allowing for reduced doses of pharmaceuticals, thus minimizing adverse effects [132]. Currently, research is focused on the synergistic interaction of herbal compounds with conventional drugs by targeting distinct biological pathways involved in disease pathology. For example, medicinal plants’ flavonoids, saponins, and alkaloids may modulate inflammatory protein pathways, influence uric acid synthesis and metabolism, and support hepatic and renal functions, leading to more effective disease management. Researchers are also exploring the potential of polyherbal formulations, which combine multiple herbs to reduce oxidative stress and promote holistic health [133].

## 6. Integrative Approaches to Managing Uric Acid and Gout

Integrating herbal and pharmacological treatments can offer significant therapeutic benefits through potential synergistic effects [134]. Herbal remedies, with their diverse phytochemical profiles, may complement conventional medications by targeting different aspects of a disease or enhancing overall therapeutic outcomes [135]. For instance, certain herbal supplements may amplify the effects of pharmacological agents or mitigate side effects. However, this integration also raises concerns about possible interactions between herbal products and conventional drugs, which could lead to adverse impacts or reduced therapeutic efficacy. Therefore, carefully evaluating these interactions is essential to avoid unfavorable outcomes [136].

In addition to medication, lifestyle modifications such as dietary changes and weight management can play a vital role in optimizing treatment efficacy [137]. These modifications can improve the body’s response to medications, reduce disease symptoms, and improve overall health. For example, dietary adjustments might influence drug metabolism or enhance nutrient absorption, while weight management can affect medication dosages and effectiveness [138].

Personalized medicine is integral to this approach, as it customizes treatment plans based on individual patient characteristics, including genetic constituents, lifestyle, and health conditions. By tailoring herbal and pharmacological treatments according to patient’s specific needs and profiles, personalized medicine can help maximize therapeutic efficacy while minimizing risks. This individualized strategy ensures that treatments are practical and safe, reducing the likelihood of adverse interactions and promoting a more holistic and patient-centered approach to healthcare.

## 7. Conclusions

Gout is an ancient inflammatory arthritis characterized by the deposition of uric acid crystals in the joints, leading to painful flare-ups. The growing prevalence of this condition underscores the need for effective management strategies that combine modern pharmacological approaches and traditional herbal remedies. Chemical medications, such as allopurinol and febuxostat, have demonstrated efficacy in lowering uric acid levels and reducing the frequency and severity of gout attacks. These drugs work by inhibiting the enzyme xanthine oxidase, which is responsible for the production of uric acid. However, these synthetic drugs can sometimes cause adverse side effects, including rashes, gastrointestinal discomfort, and liver or kidney dysfunction.

In contrast, certain herbal remedies have shown promise in managing gout, offering supplementary benefits beyond just lowering uric acid levels. Turmeric, ginger, and cherry extracts have been found to possess anti-inflammatory and antioxidant properties, which can help alleviate the pain and swelling associated with gout flare-ups. Turmeric, for instance, contains the active compound curcumin, which has been shown to inhibit the pro-inflammatory transcription factor NF-κB, thereby reducing the production of inflammatory mediators. Ginger, conversely, contains compounds like gingerol and shogaol that can suppress the activity of enzymes involved in the inflammatory response.

In conclusion, the therapeutic control of uric acid levels and gout using chemical and herbal drugs underscores the potential synergy between contemporary pharmacology and ancient therapies. Chemical medications, including allopurinol and febuxostat, efficiently lower uric acid levels, while herbal remedies, such as turmeric, ginger, and cherry extract, provide supplementary advantages, including anti-inflammatory and antioxidant effects. Subsequent research must concentrate on clinical trials that integrate these methodologies to improve effectiveness and reduce adverse effects. However, challenges in the standardization, dosage, and regulatory approval of herbal remedies remain, requiring more rigorous, controlled research to incorporate traditional knowledge into contemporary therapeutic approaches effectively.

## Figures and Tables

**Table 1:** Genetic factors contributing to gout risk [50].

Gene Function	Gene Name	Gene Product	Location
Production of uric acid	HPRT1	Hypoxanthine-guanine phosphoribosyltransferase (HGPRT)	Xq26.2-26.3
	PRPS1	Phosphoribosyl pyrophosphate synthetase 1 (PRPPS)	Xq22.3
	XOR	Xanthine oxidoreductase	2p23.1
	ALDH16A1	Aldehyde dehydrogenase 16 family A1	19q13.33
Excretion/Transport/Anion exchange/Renal drug excretion	SLC22A11	Organic anion transporter 4 (OAT4)	11q13.1
	UOX	Uricase (UOX)	1p22.1
	SLC22A12	Urate transporter 1 (URAT1)	11q13.1
	SLC22A13	Organic anion transporter 10 (OAT10)	3p22.2
	SLC2A9	Glucose transporter 9 (GLUT9)	4p16.1
	ABCG2	ATP-binding cassette subfamily G member 2 (BCRP)	4q22.1
	ABCC4	Multidrug resistance-associated protein 4 (MRP4)	13q32.1
	SLC17A1	Sodium-dependent phosphate transport protein 17A1	6p22.2
	SLC17A3	Sodium-dependent phosphate transporter 17A3	6p22.2
	SLC17A4	Sodium-dependent phosphate transporter 17A4	6p22.2
	SLC22A6	Solute carrier family 22 member 6 (OAT1)	11q13
	SLC22A8	Organic anion transporter 3 (OAT3)	11q13

**Table 2:** Uric acid-lowering medications [75,76].

Drug	Mechanism of Action	Indication	Dosage	Side Effects
Probenecid	Inhibits URAT1, reducing uric acid reabsorption in the kidneys.	Underexcretion of uric acid; adjunct to XOI if monotherapy is insufficient.	500 mg twice daily	Gastrointestinal distress, nephrolithiasis (kidney stones), hypersensitivity reactions.
Allopurinol	Xanthine oxidase inhibitor (XOI); reduces production of uric acid.	Hyperuricemia, gout, prevention of tumor lysis syndrome.	100–300 mg daily	Rash, gastrointestinal upset, liver enzyme elevation, hypersensitivity reactions.
Febuxostat	Xanthine oxidase inhibitor (XOI); reduces production of uric acid.	Chronic gout in patients who cannot tolerate allopurinol.	40–80 mg daily	Liver enzyme elevation, rash, and cardiovascular events in some patients.
Rasburicase	Uricase analog; converts uric acid into allantoin for easier excretion.	Hyperuricemia associated with tumor lysis syndrome.	0.15–0.2 mg/kg IV	Hypersensitivity, fever, gastrointestinal symptoms, anaphylaxis in rare cases.
Lesinurad	Inhibits URAT1 and OAT4 transporters, reducing uric acid reabsorption.	Used with XOI in patients not achieving target SU levels with XOI alone.	200 mg daily	Increased serum creatinine, renal events (mainly if used as monotherapy), headache.
Benzbromarone	Inhibits URAT1, promoting uric acid excretion.	Underexcretion of uric acid.	50–100 mg daily	Hepatotoxicity, gastrointestinal discomfort, liver enzyme elevation.
Arhalofenate	Dual action: Inhibits URAT1 and has anti-inflammatory effects via NLRP3 inflammasome inhibition.	Dual urate-lowering and anti-inflammatory prophylaxis.	600–800 mg daily	Gastrointestinal issues, reduced flare rates in gout patients.
Verinurad	Selective URAT1 inhibitor, used in combination with XOIs.	Adjunct to XOI therapy.	2.5–20 mg daily	Renal events and elevated serum creatinine.

**Table 3:** Comparative overview of herbal and conventional treatments for gout [112,113,114,115,116,117,118,119,120,121,122].

Treatment Type	Preparation	Composition	Effects	Action	Combination with Conventional Drugs	Dosing Protocols	Efficacy Rates (*p*-Values, CI)	Adverse Effects and Evidence Base
**Herbal Treatments**	Simiao Powder	Dried leaves of the plant Cortex Phellodendri Amurensis, Semen Coicis, Radix Achyranthis Bidentatae, Rhizoma Atractylodis Lanceae	Uric acid-lowering, immunomodulatory, and anti-inflammatory	Reduces joint discomfort, encourages uric acid excretion, and inhibits TNF-α & IL-6.	Jiawei Simiao Powder, Tongfeng Decoction, and Danxi Tongfeng Decoction are among the modified formulae.	2–3 g daily, divided doses	Efficacy Rate: 80% (*p* < 0.05) CI: 95%	Minor GI irritation reported; supported by trials (n = 100, 6 months)
	Wuwei Xiaodu Decoction	N/A (contains herbs that remove heat and are cleansing)	Eliminates heat, detoxifies, alleviates joint inflammation and discomfort	Enhances efficacy in treating acute gout arthritis when combined with colchicine	Combined with colchicine for improved efficacy against acute gout arthritis	200 mL daily, divided doses	Efficacy Rate: 76% (*p* < 0.05) CI: 90%	Nausea, minor GI upset; meta-analysis data available (n = 120, 4 months)
	Zhuye Shigao Decoction	N/A (used for clearing heat and nourishing yin)	Clears heat, nourishes yin	Significantly improves gout symptoms, reduces gastrointestinal side effects when combined with gout medications	Combined with colchicine and celecoxib to improve symptoms and reduce side effects	200 mL/day	Efficacy Rate: 65% (*p* < 0.01) CI: 95%	Dizziness, GI discomfort; backed by smaller trials (n = 50, 3 months)
	Qingre Chubi Decoction	Herba Aristolochiae Mollissimae, Radix Stephaniae Tetrandrae	Dispels wind, drains dampness, relieves pain	Reduces inflammatory markers, improves uric acid excretion	Combined with etoricoxib to treat acute gout, enhancing the therapeutic effect and reducing inflammation	100 mL 2x/day	Efficacy Rate: 72% (*p* < 0.05) CI: 95%	GI effects; contraindicated in liver impairment (meta-analysis n = 90, 5 months)
	Achyranthes Bidentata	Dried root	Uric acid-lowering, improves blood circulation	Enhances uric acid metabolism, reduces joint pain	Used alone or in combination with conventional medications	2–5 g powder daily	Efficacy Rate: 70% (*p* < 0.01) CI: 95%	Rare GI side effects; data from RCT (n = 80, 3 months)
	Gentiana Macrophylla	Root	Anti-inflammatory, relieves pain	Reduces joint inflammation, alleviates swelling, promotes uric acid excretion	Commonly used in combination with anti-gout medications	4–6 g daily	Efficacy Rate: 68% (*p* < 0.01) CI: 90%	GI upset, rare allergic reactions; supported by trials (n = 60, 4 months)
**Conventional Treatments**	NSAIDs (e.g., Indomethacin, Naproxen)	Synthetic drugs	Pain relief, anti-inflammatory	Inhibits COX enzymes, reducing prostaglandin synthesis	Often first-line treatment, may be combined with colchicine or corticosteroids	Standard dosing per drug label	Efficacy Rate: 85% (*p* < 0.01) CI: 95%	GI, cardiovascular risks; meta-analysis data (n = 200, 6 months)
	Colchicine	Synthetic drug	Reduces inflammation, pain relief	Inhibits microtubule polymerization, reducing neutrophil migration	Used alone or with NSAIDs for acute attacks	Initial high dose, reduced maintenance	Efficacy Rate: 75% (*p* < 0.01) CI: 90%	Nausea, diarrhea, GI symptoms; large trials (n = 150, 5 months)
	Corticosteroids	Synthetic drugs	Pain relief, anti-inflammatory	Inhibits inflammatory mediators and immune response	Sometimes combined with NSAIDs for severe cases	Standard dose adjustments per protocol	Efficacy Rate: 82% (*p* < 0.01) CI: 90%	Immunosuppression risks; meta-analysis (n = 180, 6 months)
	Biologics (e.g., Anakinra, Canakinumab)	Synthetic IL-1 inhibitors	Reduces inflammation in refractory cases	Blocks IL-1-mediated inflammation pathways	Reserved for patients unresponsive to standard treatments	Administered per physician guidance	Efficacy Rate: 78% (*p* < 0.01) CI: 90%	Infection risk; limited data available, small trials (n = 30, 1 year)

**Table 4:** Overview of phytochemicals and their mechanisms in uric acid reduction [126].

Class	Compound	Source Plant	Mechanism of Action	Effects on Uric Acid
**Flavonoids**	Quercetin	*Allium cepa* (onions), *Malus domestica* (apples)	Inhibits xanthine oxidase (XOD) and reduces inflammatory markers (IL-1β, NLRP3).	Lowers uric acid levels, IC_50_ of 2.74 µmol/L for XOD inhibition, a potential treatment for HUA.
	Kaempferol	*Brassica oleracea* (kale), *Ginkgo biloba*	It inhibits XOD and regulates uric acid transporters (URAT1).	Reduces serum uric acid levels and mitigates hyperuricemia symptoms.
	Luteolin	*Citrus spp*. (citrus fruits), *Apium graveolens* (celery)	Competitive XOD inhibition with a high binding affinity (2.38 × 10^−6^ mol/L).	Effective in lowering uric acid levels in clinical trials and animal studies.
	Isorhamnetin	*Hippophae rhamnoides* (sea buckthorn)	Inhibits hepatic XOD activity and reduces serum uric acid levels.	Significant uric acid reduction in animal models, potential therapeutic agent.
**Phenolic Acids**	Chlorogenic Acid	*Coffea arabica* (coffee), *Eugenia uniflora* (surinam cherry)	Inhibits XOD activity, reduces uric acid production, and modulates inflammatory pathways.	Decreases serum uric acid levels and inhibits XOD activity.
	Caffeic Acid	*Salvia officinalis* (sage), *Coffea arabica* (coffee)	Down-regulates URAT1 and GLUT9, up-regulates OAT1, competitive XOD inhibition.	Reduces uric acid levels and manages hyperuricemia through modulation
**Saponins**	Ginsenosides	*Panax ginseng*	Anti-inflammatory properties that may indirectly support uric acid reduction.	Indirectly influences uric acid levels through overall health improvement.
**Alkaloids**	Vindoline	*Vinca rosea* (periwinkle)	Broader therapeutic actions that impact metabolic pathways.	

## Data Availability

Data analyzed in this review study are cited from published articles.
